# Real-world characteristics and outcomes of patients with high-risk and non-high-risk smoldering multiple myeloma using the Flatiron Health database

**DOI:** 10.1038/s41408-024-01170-z

**Published:** 2024-12-05

**Authors:** S. Vincent Rajkumar, María-Victoria Mateos, Marcy Schaeffer, Xiwu Lin, Sacheeta Bathija, Niodita Gupta-Werner, Annette Lam, Robin Carson, Robyn Dennis, Shuchita Kaila, Kathryn Matt, Joana Duran, Sagar Lonial

**Affiliations:** 1 Mayo Clinic, Rochester, MN USA; 2 Hospital Universitario de Salamanca, Instituto de Investigación Biomédica de Salamanca (IBSAL), Centro de Investigación del Cancer (IBMCC-USAL, CSIC), Salamanca, Spain; 3 Janssen Pharmaceutica NV, Beerse, Belgium; 4 Janssen Global Services, Horsham, PA USA; 5 Janssen Global Services, Raritan, NJ USA; 6 Janssen Scientific Affairs, Horsham, PA USA; 7 Janssen Research & Development, Wayne, PA USA; 8 Emory University School of Medicine, Atlanta, GA USA

**Keywords:** Myeloma, Risk factors

## Abstract

This study aimed to provide real-world evidence on progression risk in patients with high-risk smoldering multiple myeloma (SMM). This retrospective, observational study leveraged data from the Flatiron Health database. Eligible patients had SMM and relevant measures to apply Mayo 2018, International Myeloma Working Group (IMWG) 2020, and AQUILA trial risk criteria. Time to progression to active MM (TTP), progression or death (PFS), and death or progression on first-line MM therapy (PFS2) were evaluated using Kaplan–Meier methods and multivariate Cox regression models adjusted for age, Charlson Comorbidity Index, and time from SMM diagnosis to risk classification date. Across the three risk models (Mayo 2018, IMWG 2020, and AQUILA trial), high-risk patients with SMM had 3.0–4.0 times the risk of TTP, 2.1–3.5 times the risk of PFS, and 1.7–3.2 times the risk of PFS2 versus non-high-risk patients (*p* < 0.001 for all comparisons). Similar results were observed when patients with early treatment, early progression, and/or bone disease were excluded. This study demonstrates that high-risk patients with SMM have worse prognoses than non-high-risk patients, regardless of the criteria used, and highlights a need for early intervention testing.

## Introduction

Smoldering multiple myeloma (SMM) is an asymptomatic clonal plasma cell disorder that is a precursor to active multiple myeloma (MM) [1]. SMM is a heterogenous entity comprising patient populations with biology that resembles monoclonal gammopathy of undetermined significance but with a higher risk of progression to active MM [2–4]. Within 5 years of diagnosis, it is estimated that 51% of patients with SMM will progress to active MM, increasing to 66% over 10 years [5]. However, progression occurs more rapidly in some patients than others [6], and the identification of those patients with SMM who are at high risk of progression to active MM allows for disease management to be tailored specifically to their needs. Currently, there are no approved treatments for patients with SMM. While the standard of care is currently a “watch and wait” approach with regular follow-up visits until symptoms of active MM emerge, those patients with a high risk of progression should be encouraged to participate in clinical trials [2, 7, 8]. On the basis of lower-level evidence, single-agent lenalidomide is considered a treatment option by the NCCN Clinical Practice Guidelines in Oncology (NCCN Guidelines®) only in certain carefully selected high-risk patients with SMM [9].

Several approaches have been proposed to identify patients at high risk for progression to active MM. The ongoing randomized, open-label, phase 3 AQUILA trial is investigating the efficacy and safety of subcutaneous daratumumab versus active monitoring in high-risk patients with SMM. High risk was classified in AQUILA as bone marrow plasma cell percentage (BMPC%) ≥10% and one or more of the following: serum M-protein ≥30 g/L, IgA SMM, immunoparesis with reduction of two uninvolved Ig isotypes, serum involved:uninvolved free light chain ratio (FLCr) ≥ 8 to <100 or clonal BMPCs >50% to <60% with measurable disease, and Eastern Cooperative Oncology Group performance status ≤1 [10]. A model developed by the Mayo Clinic (Mayo 2018 model, also known as 20/2/20), includes three criteria: BMPC% >20%, serum M-protein >2 g/dL, and serum FLCr >20 (Table 1) [11]. The Mayo 2018 model has been widely employed due to its generalizability and ease of use [12]. The International Myeloma Working Group (IMWG) 2020 model was developed to further improve upon the robustness of the Mayo 2018 stratification criteria by refining the risk factors to include genetic abnormalities (translocation t(4;14), t(14;16), 1q gain, del13q, and monosomy 13) (Table 1) [13]. Based on the number of risk factors, patients can be separated into four groups (0 = low risk, 1 = intermediate-low risk, 2 = intermediate risk, and ≥3 = high risk), with a corresponding 2-year risk of progression of 6%, 23%, 46%, and 63%, respectively. The criteria for defining high-risk SMM is constantly evolving and being refined. Presently, no risk-stratification model has been adopted consistently, each having its own advantages and limitations.

**Table 1:** High-risk classification at SMM diagnosis.

Risk component	Mayo clinic (at least 2 of + components)	IMWG 2020 (at least 3 of + components)	AQUILA trial (BMPC% and at least 1 of + components)
Clonal BMPC%	+ >20% to <60%	+ >20%	X ≥10% + >50% to <60%
Serum M-protein	+ >20 g/L	+ >20 g/L	+ ≥30 g/L
Immunoglobulin isotype and immunoparesis	-	-	+ IgA
FLCr	+ >20 and <100	+ >20	+ ≥8 and <100
t(4;14), t(14;16), +1q, and/or del13q/monosomy 13 abnormality	-	+	-

*BMPC%* bone marrow plasma cell percentage, *IMWG* International Myeloma Working Group, *FLCr* serum free light chain ratio, *SMM* smoldering multiple myeloma. X: component required; +: at least 1,2,3 component(s) required; -: component not part of risk criteria. For the Mayo Clinic criteria, low-risk patients had no components; for IMWG criteria, low-risk patients had 1 or no components; and for the AQUILA trial criteria, low-risk patients did not satisfy the criteria. For the Mayo Clinic criteria, intermediate-risk patients had 1 of the + components, and for the IMWG 2020 criteria, intermediate-risk patients had 2 of the + components.

The objective of this real-world study was to evaluate and compare the characteristics and outcomes of patients with SMM stratified into high-risk and non-high-risk categories using the Mayo 2018, IMWG 2020, and AQUILA trial criteria.

## Methods

### Study population

This retrospective, observational study leveraged data from the Flatiron Health database. This nationwide oncology practices registry includes deidentified patient data from electronic health records from approximately 280 cancer clinics in the United States and has been used extensively to generate real-world evidence on various cancers, including MM [14–16]. Patients were included in the SMM cohort if they had an International Classification of Disease (ICD) diagnosis of MM (ICD-9 203.0x or ICD-10 C90.0x, C90), and then patient charts were manually reviewed by Flatiron’s data abstractors, with oversight from Flatiron’s oncology team for confirmation of SMM diagnosis and date; ≥2 visits recorded in the Flatiron Health database on or after 1 January 2011; date of SMM diagnosis between 1 January 2011 and 1 August 2018, inclusive to allow for the potential for at least 3 years of follow-up (through 31 August 2021); available measures to apply the Mayo 2018, IMWG 2020, and/or AQUILA trial risk-stratification criteria.

### Risk evaluation

Patients were stratified by risk using the Mayo 2018, IMWG 2020, and AQUILA trial models (Table 1). The risk components evaluated across the three models included clonal BMPC, serum M-protein, immunoglobulin isotype and immunoparesis, FLCr, and genetic abnormality (t(4;14), t(14;16), +1q, and/or del13q/monosomy 13 abnormality). Patients qualified as high risk if they had at least the minimum number of appropriate risk components for the criteria measured and were not required to have all the individual risk components for that particular model; patients of low and intermediate risk were also tested for the appropriate risk components (Table 1). For patients with all available risk factors measured within 60 days of SMM diagnosis, these factors were used to define risk, and their index date was set to the date of SMM diagnosis. For patients without all risk factors measured within 60 days of SMM diagnosis, measurements for each risk factor closest to the date of SMM diagnosis were considered. The index date for these patients was set to the date of the latest risk factor measurement closest to SMM diagnosis, and all other risk factor measures were chosen to be closest to this date.

Patients stratified as non-high-risk with the Mayo and IMWG criteria comprised those who were low and intermediate risk (definition in Table 1), and intermediate-risk patients for IMWG included patients classified as intermediate-low or intermediate risk. Patients who were classified as non-high risk by the IMWG criteria were required to have a test performed for all genetic markers.

### Outcomes

Patients were considered to have progressed to active MM if they had an ICD code for MM and a physician statement in the patient’s record confirming the MM diagnosis. Time to progression (TTP) was defined as the time from index date to MM diagnosis (patients who died were censored at the time of death). Progression-free survival (PFS) was the time from index date to MM diagnosis or the date of death, whichever occurred first, and PFS2 was the time from index date to the date of documented death or progression on first-line (1 L) treatment for active MM (MM treatment as identified in the Flatiron Health database), whichever occurred first. Progression on 1 L treatment for MM was defined based on an enhanced M spike and structured FLC laboratory values or the date of starting a second-line treatment, whichever occurred first. Patients were censored at the end of follow-up (last observation date) if they did not have the event for the corresponding endpoint. The proportion of patients receiving 1 L MM treatment was also assessed.

Four separate sensitivity analyses were conducted to test the robustness of the real-world collection of data records. In the first of these sensitivity analyses, the regression models were additionally adjusted for race, creatinine, and hemoglobin levels. A second sensitivity analysis was performed to exclude patients who may potentially be patients with MM but who were classified as SMM. In this analysis, patients were excluded who received 1 L treatment for MM within 60 days of SMM diagnosis, had progression within 90 days of SMM diagnosis, and/or had bone disease diagnosis within 60 days of SMM diagnosis. As progression may be missed in those patients who had a long gap between their last observation and death, the final two sensitivity analyses included censoring patients who died more than 180 days or more than 365 days after the last observation in the Flatiron Health database.

### Statistical analyses

Primary analyses were based on the entire SMM cohort with an assigned risk level. The relationships between risk level and clinical outcomes were analyzed using Kaplan–Meier methods and multivariate Cox regression models adjusted for age, Charlson Comorbidity Index (CCI), and the time from SMM diagnosis to the index date. Results were reported as hazard ratios (HRs) with 95% CIs and *P* values.

All statistical analyses and graphical interpretation of the results were performed with SAS 9.4 (SAS Institute, Cary, NC, USA) and R version 4.2.1 (R Foundation for Statistical Computing, Vienna, Austria).

## Results

### Study population and risk-stratification

The Flatiron registry included data from 1001 patients with SMM; five patients were initially excluded due to data abnormalities. Among the remaining 996 patients, the median time from SMM diagnosis to the end of observation or death was 54.1 months. Overall, 412 patients progressed to MM, and 272 deaths were reported (49.6% were prior to progression).

Based on available data to apply Mayo 2018, IMWG 2020, and AQUILA trial criteria, of the 996 patients with SMM, 761 (76.4%), 261 (26.2%), and 707 (71.0%) were risk-stratified and of these 162/761 (21.3%), 103/261 (39.5%), and 431/707 (61.0%) were classified as high risk and 599/761 (78.7%), 158/261 (60.5%), and 276/707 (39.0%) as non-high risk, respectively (Table 2).

**Table 2:** Risk-stratification for the overall population.

Stratification model	Risk classification, *n* (%) (*N* = 996)		
	High risk	Non-high risk	Not applicable
Mayo 2018	162 (16.3)	599 (60.1)	235 (23.6)
IMWG 2020	103 (10.3)	158 (15.9)	735 (73.8)
AQUILA Trial	431 (43.3)	276 (27.7)	289 (29.0)

*IMWG* International Myeloma Working Group.

Patient demographics were similar across risk groups and stratification criteria (Table 3 and Supplementary Tables 1 and 2). Median age was 70 years, and the majority of the study population were white (61%) and had a CCI of 0 (71%).

**Table 3:** Baseline characteristics in risk-stratified patients with SMM.

Characteristic	Mayo 2018 criteria		IMWG 2020 criteria		AQUILA trial criteria		All Patients
	High risk *n* = 162	Non-high risk^a^ *n* = 599	High risk *n* = 103	Non-high risk^a^ *n* = 158	High risk *n* = 431	Non-high risk *n* = 276	*N* = 996
Age,^b^ years							
Mean (SD)	67.9 (9.3)	67.4 (9.9)	67.3 (9.1)	68.1 (10.3)	67.2 (9.8)	67.9 (9.6)	67.6 (9.8)
Median (min, max)	70.0 [37.0, 82.0]	69.0 [29.0, 82.0]	69.0 [41.0, 82.0]	70.0 [36.0, 82.0]	70.0 [36.0, 82.0]	69.0 [36.0, 82.0]	70.0 [29.0, 82.0]
Male sex, *n* (%)	93 (57.4%)	310 (51.8%)	57 (55.3%)	74 (46.8%)	231 (53.6%)	139 (50.4%)	520 (52.2%)
Race and ethnicity, *n* (%)							
White	109 (67.3%)	369 (61.6%)	71 (68.9%)	100 (63.3%)	272 (63.1%)	173 (62.7%)	612 (61.4%)
Black/African American	17 (10.5%)	111 (18.5%)	14 (13.6%)	27 (17.1%)	60 (13.9%)	51 (18.5%)	165 (16.6%)
Hispanic/Latino	1 (0.6%)	8 (1.3%)	0 (0%)	0 (0%)	5 (1.2%)	3 (1.1%)	11 (1.1%)
Asian	2 (1.2%)	9 (1.5%)	2 (1.9%)	3 (1.9%)	9 (2.1%)	3 (1.1%)	15 (1.5%)
Other race	21 (13.0%)	67 (11.2%)	9 (8.7%)	23 (14.6%)	52 (12.1%)	30 (10.9%)	117 (11.7%)
Missing	12 (7.4%)	35 (5.8%)	7 (6.8%)	5 (3.2%)	33 (7.7%)	16 (5.8%)	76 (7.6%)
ECOG score at the time of SMM diagnosis, *n* (%)							
0	44 (27.2%)	165 (27.5%)	32 (31.1%)	58 (36.7%)	111 (25.8%)	75 (27.2%)	264 (26.5%)
1	15 (9.3%)	72 (12.0%)	10 (9.7%)	24 (15.2%)	48 (11.1%)	28 (10.1%)	118 (11.8%)
2	3 (1.9%)	21 (3.5%)	2 (1.9%)	6 (3.8%)	8 (1.9%)	12 (4.3%)	30 (3.0%)
3	0 (0%)	1 (0.2%)	0 (0%)	0 (0%)	1 (0.2%)	1 (0.4%)	2 (0.2%)
Missing	100 (61.7%)	340 (56.8%)	59 (57.3%)	70 (44.3%)	263 (61.0%)	160 (58.0%)	582 (58.4%)
Charlson comorbidity index, *n* (%)							
0	113 (69.8%)	426 (71.1%)	72 (69.9%)	113 (71.5%)	316 (73.3%)	189 (68.5%)	709 (71.2%)
1	8 (4.9%)	47 (7.8%)	3 (2.9%)	20 (12.7%)	25 (5.8%)	23 (8.3%)	63 (6.3%)
2	34 (21.0%)	105 (17.5%)	25 (24.3%)	23 (14.6%)	77 (17.9%)	52 (18.8%)	189 (19.0%)
3–6	7 (4.3%)	21 (3.5%)	3 (2.9%)	2 (1.3%)	13 (3.0%)	12 (4.3%)	35 (3.5%)
Last laboratory value prior to index date, mean (SD)							
SCr, mg/dL	1.1 (0.5)	1.2 (0.9)	1.1 (0.5)	1.2 (1.1)	1.1 (0.7)	1.2 (0.9)	1.1 (0.8)
Hemoglobin, g/dL	12.8 (1.6)	12.8 (1.9)	12.9 (1.4)	12.8 (1.8)	12.9 (1.8)	12.8 (1.8)	12.7 (1.8)
SCa, mg/dL	9.3 (0.5)	9.4 (0.6)	9.4 (0.5)	9.4 (0.6)	9.4 (0.5)	9.4 (0.6)	9.4 (0.6)
LDH, U/L	250 (168)	203 (113)	214 (125)	216 (129)	192 (101)	224 (143)	206 (121)

^a^Includes patients stratified to low or intermediate risk.

^b^Age at diagnosis.

*ECOG* Eastern Cooperative Oncology Group, *LDH* lactate dehydrogenase, *SCa* serum calcium, *SCr* serum creatinine, *SD* standard deviation, *SMM* smoldering multiple myeloma.

### Patient outcomes by risk

High-risk patients with SMM were at greater risk for all evaluated outcomes versus non-high-risk patients. TTP, PFS, and PFS2 were shorter in high-risk versus non-high-risk patients in each of the three models (Table 4).

**Table 4:** Outcomes in risk-stratified patients with SMM.

Endpoint	High-risk median (95% CI), months^a^	Non-high risk median (95% CI), months^b,c^	Hazard ratio (95% CI)	*p* value
TTP				
Mayo 2018	13.0 (11.0, 19.3)	90.1 (77.0, NE)	3.9 (3.1, 4.9)	*p* < 0.0001
IMWG 2020	16.4 (11.0, 22.0)	80.0 (79.1, NE)	4.0 (2.9, 5.7)	*p* < 0.0001
AQUILA trial	35.3 (29.6, 43.1)	NE (NE, NE)	3.0 (2.3, 3.9)	*p* < 0.0001
PFS				
Mayo 2018	12.9 (11.0, 19.0)	56.7 (51.5, 69.0)	3.0 (2.4, 3.7)	*p* < 0.0001
IMWG 2020	16.0 (11.0, 21.0)	63.5 (39.9, NE)	3.5 (2.5, 4.8)	*p* < 0.0001
AQUILA trial	30.0 (22.8, 35.7)	71.3 (61.4, NE)	2.1 (1.7, 2.7)	*p* < 0.0001
PFS2				
Mayo 2018	35.9 (30.0, 44.3)	71.0 (63.5, 76.4)	2.3 (1.8, 2.9)	*p* < 0.0001
IMWG 2020	33.5 (27.4, 45.6)	84.2 (63.5, NE)	3.2 (2.2, 4.6)	*p* < 0.0001
AQUILA trial	52.1 (47.3, 60.1)	74.1 (69.0, NE)	1.7 (1.3, 2.1)	*p* < 0.0001

^a^Mayo 2018, *n* = 163; IMWG 2020, *n* = 105; AQUILA trial, *n* = 432.

^b^Includes patients stratified to low or intermediate risk.

^c^Mayo 2018, *n* = 601; IMWG 2020, *n* = 296; AQUILA trial, *n* = 278.

*CI* confidence interval, *NE* not estimable, *MM* multiple myeloma, *PFS* progression or death, *PFS2* progression or death on first-line MM treatment, *SMM* smoldering multiple myeloma, *TTP* time to progression.

**Fig. 1:**
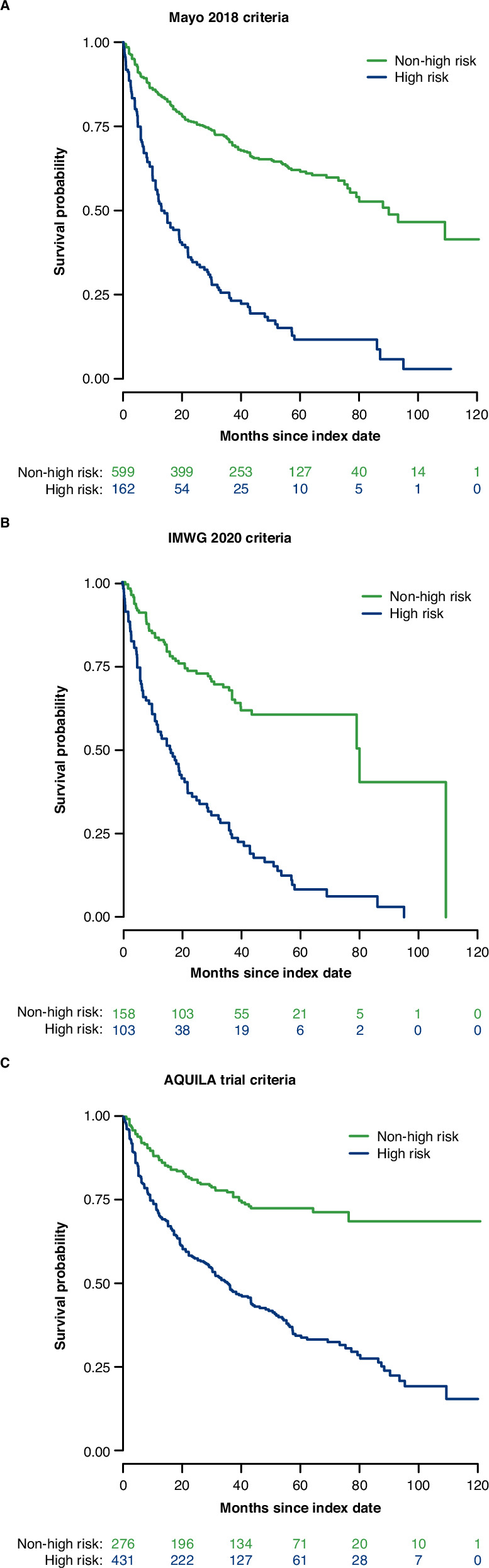
Progression to MM by risk criteria. **A** Mayo 2018 criteria. **B** IMWG 2020 criteria. **C** AQUILA trial criteria. IMWG International Myeloma Working Group, MM multiple myeloma.

Across the three models, the risk of progression to MM (TTP) was 3.0–4.0 times higher with high-risk than non-high-risk patients (HR [95% CI]: Mayo 2018 criteria, 3.9 [3.1–4.9], IMWG 2020 criteria, 4.0 (2.9–5.7), and AQUILA trial criteria 3.0 [2.3–3.9]; Table 4 and Fig. 1) adjusted for age, CCI, and time between SMM diagnosis and risk classification.

**Fig. 2:**
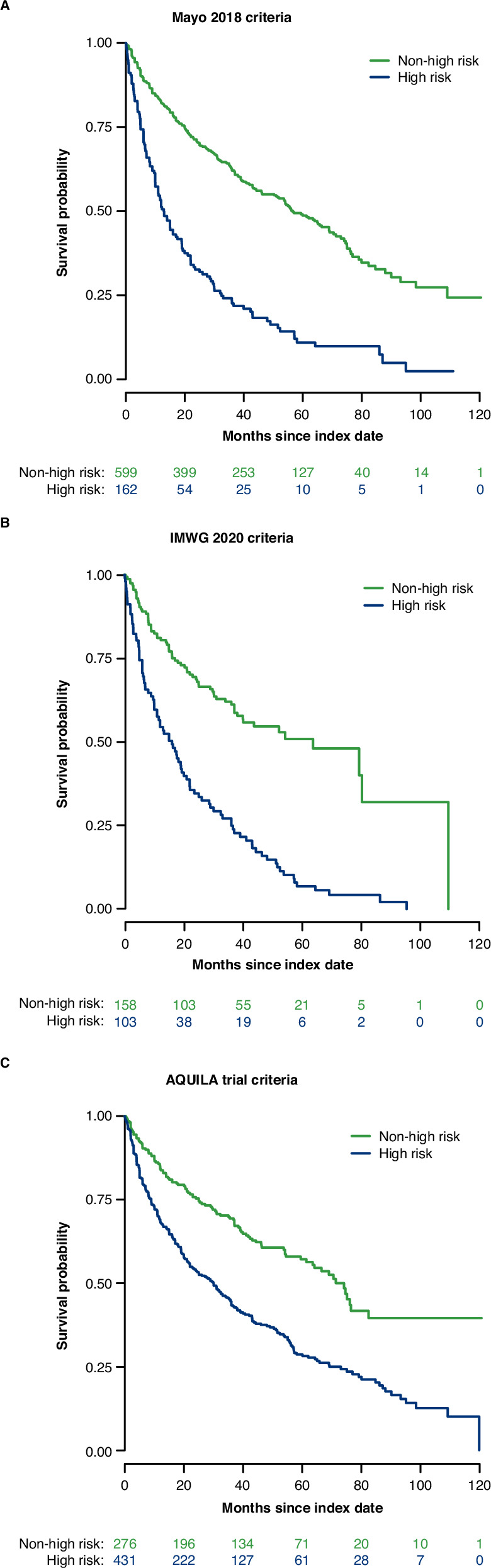
Progression to MM or death by risk criteria. **A** Mayo 2018 criteria. **B** IMWG 2020 criteria. **C** AQUILA trial criteria. IMWG International Myeloma Working Group, MM multiple myeloma.

The risk of progression to MM or death (PFS) was 2.1–3.5 times greater in high-risk compared with non-high-risk patients (HR [95% CI]: Mayo 2018 criteria, 3.0 [2.4–3.7], IMWG 2020 criteria, 3.5 (2.5–4.8) and AQUILA trial criteria, 2.1 [1.7–2.7]; Table 4 and Fig. 2)) adjusted for age, CCI, and time between SMM diagnosis and risk classification.

**Fig. 3:**
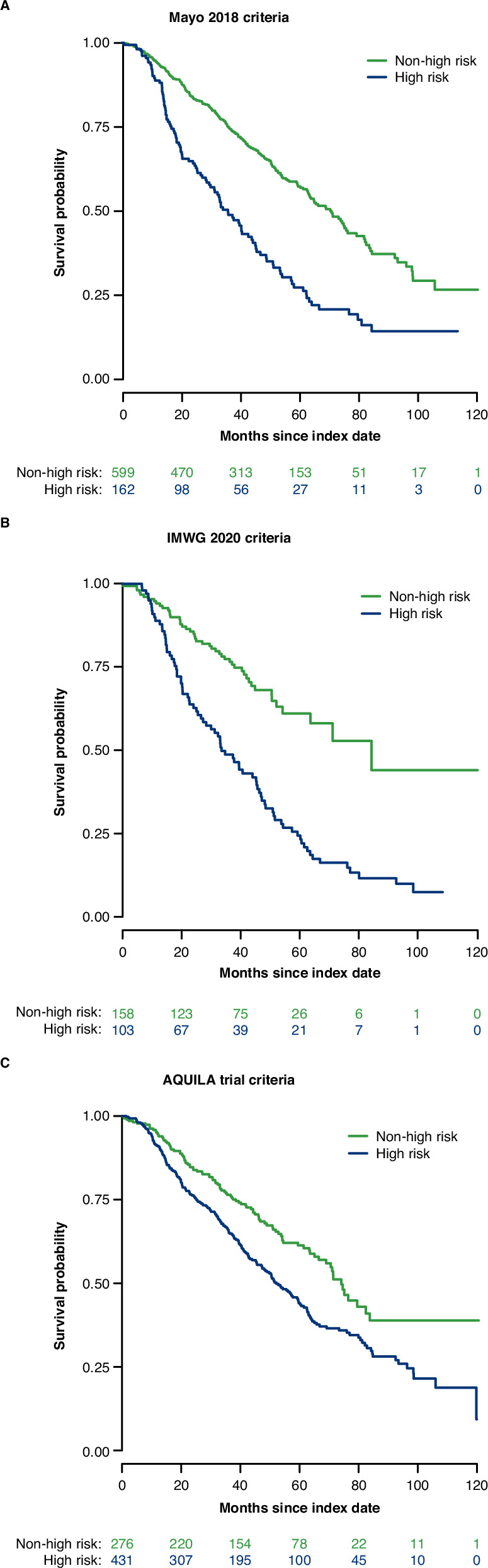
Progression on first-line MM treatment or death by risk criteria. **A** Mayo 2018 criteria. **B** IMWG 2020 criteria. **C** AQUILA trial criteria. IMWG International Myeloma Working Group, MM multiple myeloma.

A greater proportion of high-risk patients received 1 L treatment for MM during the follow-up compared with the non-high-risk patients: Mayo 2018 criteria, *n* = 121/162 (74.7%) and *n* = 193/599 (32.2%); IMWG 2020 criteria, *n* = 87/103 (84.5%) and *n* = 55/158 (34.8%); AQUILA trial criteria, *n* = 230/431 (53.4%) and *n* = 66/276 (23.9%), respectively. The risk of death or progression after receiving 1 L (PFS2) treatment for MM was 1.7–3.2 times higher in high-risk compared with non-high-risk patients (HR [95% CI]: Mayo 2018 criteria, 2.3 [1.8–2.9], IMWG 2020 criteria, 3.2 (2.2–4.6), and AQUILA trial criteria, 1.7 [1.3–2.1]; Table 4 and Fig. 3) adjusted for age, CCI, and time between SMM diagnosis and risk classification.

### Sensitivity analyses

Results from the sensitivity analyses were similar to the primary analysis. When data were adjusted for age, CCI, time between SMM diagnosis and index date, race, creatinine, and hemoglobin across the three high-risk criteria models, high-risk patients had a 3.2–4.9, 2.4–3.9, and 2.1–4.0 higher risk of TTP, PFS, and PFS2, respectively, versus non-high-risk patients (Table 5).

**Table 5:** Outcomes in risk-stratified patients with SMM (high-risk versus non-high-risk patients)-sensitivity analyses.

Endpoint (sensitivity analysis)	TTP Hazard ratio (95% CI)	PFS Hazard ratio (95% CI)	PFS2 Hazard ratio (95% CI)
Adjusted for age, CCI, time between SMM diagnosis and index date, race, creatinine, and hemoglobin			
Mayo 2018	4.9 (3.7, 6.4)	3.6 (2.8, 4.6)	2.9 (2.2, 3.7)
IMWG 2020	4.5 (3.0, 6.6)	3.9 (2.7, 5.6)	4.0 (2.7, 6.0)
AQUILA trial	3.2 (2.4, 4.5)	2.4 (1.8, 3.0)	2.1 (1.6, 2.7)
In patients without early treatment, without early progression, and/or bone disease			
Mayo 2018	4.0 (3.1, 5.1)	3.1 (2.4, 3.8)	2.2 (1.7, 2.8)
IMWG 2020	4.6 (3.1, 6.7)	3.8 (2.7, 5.4)	3.3 (2.2, 4.8)
AQUILA trial	3.3 (2.4, 4.5)	2.3 (1.8, 2.9)	1.7 (1.3, 2.3)
Patients censored who died >180 days after last observation			
Mayo 2018	-	3.2 (2.6, 4.0)	2.5 (2.0, 3.2)
IMWG 2020	-	3.6 (2.6, 5.0)	3.5 (2.4, 5.0)
AQUILA trial	-	2.3 (1.9, 3.0)	1.8 (1.4, 2.3)
Patients censored who died >365 days after last observation			
Mayo 2018	-	3.1 (2.5, 3.8)	2.4 (1.9, 3.0)
IMWG 2020	-	3.6 (2.6, 4.9)	3.4 (2.3, 4.9)
AQUILA trial	-	2.3 (1.8, 2.8)	1.7 (1.3, 2.2)

*CCI* Charlson Comorbidity Index, *IMWG* International Myeloma Working Group, *MM* multiple myeloma, *PFS* progression or death, *PFS2* progression or death on first-line MM treatment, *SMM* smoldering multiple myeloma, *TTP* time to progression.

When excluding patients with possible MM (early treatment, early progression, and/or bone disease), high-risk patients had a 3.3–4.6, 2.3–3.8, and 1.7–3.3 higher likelihood of TTP, PFS, and PFS2, respectively, versus non-high-risk patients across the three high-risk criteria models (Table 5).

Similar results were seen for PFS and PFS2 across the three models when data were censored from patients who died more than 180 days (HR: 2.4–3.7 and 1.9–3.7, respectively) and 365 days (HR: 2.3–3.6 and 1.8–3.6, respectively) after the last observation (Table 5).

## Discussion

SMM, an asymptomatic precursor condition of MM, has significant heterogeneity in disease progression [17]. The paradigm shift in strategies to manage SMM in the last decade has highlighted the importance of early identification of patients with SMM at the greatest risk of progression to active MM [18, 19].

While the role of treatment in high-risk SMM is being evaluated in various clinical trials, interpretation of results is complicated by variations in the risk-stratification criteria used [7]. Several risk-stratification models have been developed, including the AQUILA trial [10], Mayo 2018 [11], IMWG 2020 [13]. While accurately characterizing patients with high-risk SMM allows for appropriate care and management of these patients, identifying patients with low-risk SMM also avoids oversurveillance and overtreatment [20].

Historically, the standard of care for SMM has been observation until patients develop active MM, regardless of their risk status [2, 7, 8]. However, results from two randomized controlled clinical trials in high-risk patients with SMM support the benefit of early therapy with lenalidomide, alone and in combination with dexamethasone, in reducing the risk of progression to active MM [21, 22].

This study leveraged data from the Flatiron Health database to assess baseline characteristics and outcomes in patients with SMM who were stratified by their risk for progression to active MM. The IMWG 2020 criteria build upon the Mayo 2018 criteria to further characterize patients as high risk through the incorporation of genetic factors, including t(4;14), t(14;16), +1q, and/or del13q/monosomy 13 abnormality [13]. The use of multiple criteria from several models to stratify risk enhanced the robustness of our results as it considered a wide array of factors comprehensively, such as clonal BMPC; serum M-protein; immunoglobulin isotype and immunoparesis; FLCr; and t(4;14), t(14;16), +1q, and/or del13q/monosomy 13 abnormality.

Depending on the stratification criteria used, 21.3%–61.0% of patients with SMM in the Flatiron Health database who could be risk-stratified were identified as having a high risk of progression to active MM. The variation in the proportion of patients who were identified as high-risk could be due to the IMWG 2020 criteria requiring the inclusion of genetic abnormalities as a stratification criterion, which is often unavailable in real-world databases. The inclusion of such information would require real-world databases to provide information not only on the receipt of the relevant genetic testing but also on the test results, whether negative or positive. While each of the three risk-stratification models used different approaches to identify high-risk patients, TTP, PFS, and PFS2 in these patients were generally similar, although time to each of these outcomes was somewhat longer in patients stratified using the AQUILA trial criteria at data cut-off. This may have been the result of the broader definition of high risk with this model, and the model may require further optimization to provide the pertinent treatment to the appropriate patients. High-risk patients with SMM were 3.2–4.9 times more likely than non-high-risk patients to progress to MM, 2.4–3.9 times more likely to progress to MM or die, and 2.1–4.0 times more likely to die or progress on 1 L MM treatment. The consistency of outcomes between the risk models provides additional confidence to these findings. Similar results have been reported previously by Visram et al., who assessed outcomes in patients who were risk-stratified using the Mayo 2018 and IMWG 2020 criteria up to 5 years post-diagnosis. In addition, a three times higher risk of progression was reported in patients who evolved to a higher-risk category during the follow-up period compared with patients with an unchanged or decreased risk category [19]. More recently, the Mayo 2018 criteria were used to risk stratify patients with SMM from the Czech Myeloma Group Registry of Monoclonal Gammopathies, and results of that study demonstrated that patient outcomes, including PFS and PFS2, were significantly worse in high-risk versus non-high-risk patients [23].

Similar results were seen in the sensitivity analyses wherein patients were adjusted for age, CCI, time between SMM diagnosis and index data, race, creatinine, and hemoglobin levels, and when data were censored from patients who died more than 180 days and 365 days after the last observation. It was considered a possibility that some patients who were classified as SMM in the Flatiron Health database may have had MM. However, when these patients were excluded in the sensitivity analyses (had received early treatment, had early progression, and/or had bone disease diagnosis), consistent results were observed.

This study does have a few limitations. Firstly, the Flatiron database does not systematically capture risk factors over time. While a small number of patients had received treatment for SMM, this information was not curated; SMM-specific treatments were not provided. As such, no definitive conclusions can be drawn about SMM treatment. In addition, the cause of death was unavailable. Finally, there was a high proportion of patients with SMM with data in the Flatiron database who were not able to be classified, and, as a result, these data may not fully represent the entirety of this patient population.

Each risk-stratification model evaluated in this study identifies a cohort of patients with SMM with an increased risk of progression to active MM and patients who overlap across the models. As has been stated previously, this is not considered a limitation. Patients identified as high risk by any of these models (e.g. IMWG identified high-risk as patients with 50% progression risk at 2 years from diagnosis based on the levels of M-protein, BMPC infiltration, or sFLC ratio) should be considered to have an increased risk of progression to active MM. Even though the models assessed in this study define high risk differently and may not be completely comparable, the benefit of the parameters used in the risk-stratification models is that they are widely available and can be used in practice and in clinical trials worldwide. We expect that models predicting risk in patients with SMM will continue to evolve, and as they do so, patients will need to be tested according to these criteria to be captured in real-world studies.

To our knowledge, this is the first real-world study evaluating risk in patients with SMM using the criteria defined in the ongoing AQUILA trial in comparison with other commonly used models. The AQUILA criteria were able to distinguish well between patients with and without a high risk of progression, and the results suggest a need for intervention in high-risk patients.

## Conclusions

Irrespective of the stratification criteria, these data demonstrate that high-risk patients with SMM are likely to have poorer prognoses than non-high-risk patients and emphasize a need to investigate early interventions for this patient population. Agreement on the consistent use of risk-stratification models will aid in comparisons between clinical and real-world studies and will help identify which patients would benefit most from early intervention, as reflected by improved patient survival and quality of life.

## Supplementary information

Supplementary Table 1. Baseline characteristics in patients with SMM risk-stratified by Mayo 2018 criteria.

Supplementary Table 2. Baseline characteristics in patients with SMM risk-stratified by IMWG 2020 criteria.

## Data Availability

The datasets generated during and/or analyzed during the current study are available from the corresponding author upon reasonable request. The real-world data were made available by Flatiron Health, Inc. and used under license for the current study and so are not publicly available. Other researchers should contact Flatiron Health, Inc. (https://flatiron.com).
