# Supplementary material for: Real-world characteristics and outcomes of patients with high-risk and non-high-risk smoldering multiple myeloma using the Flatiron Health database
Source: Blood Cancer J. 2024 Dec 5;14(1):215. doi: 10.1038/s41408-024-01170-z (PMC11621302; doi:10.1038/s41408-024-01170-z)
Supplement: Supplementary file 1 — Supplementary Table 1. Baseline characteristics in patients with SMM risk-stratified by Mayo 2018 criteria. [file 41408_2024_1170_MOESM1_ESM.docx]

**Supplementary Table 1.** Baseline characteristics in patients with SMM risk stratified by Mayo 2018 criteria.

| **Characteristic** | **High risk**  ***n* = 162** | **Intermediate risk**  ***n* = 251** | **Low risk**  ***n* = 348** |
| --- | --- | --- | --- |
| Age^a^, years |  |  |  |
| Mean (SD) | 67.9 (9.3) | 67.2 (10.3) | 67.6 (9.7) |
| Median (min, max) | 70.0 (37.0, 82.0) | 70.0 (29.0, 82.0) | 69.0 (36.0, 82.0) |
| Male sex, *n* (%) | 93 (57.4%) | 136 (54.2%) | 174 (50.0%) |
| Race and ethnicity, *n* (%) |  |  |  |
| White | 109 (67.3%) | 159 (63.3%) | 210 (60.3%) |
| Black/African American | 17 (10.5%) | 47 (18.7%) | 64 (18.4%) |
| Hispanic/Latino | 1 (0.6%) | 4 (1.6%) | 4 (1.1%) |
| Asian | 2 (1.2%) | 3 (1.2%) | 6 (1.7%) |
| Other race | 21 (13.0%) | 28 (11.2%) | 39 (11.2%) |
| Missing | 12 (7.4%) | 10 (4.0%) | 25 (7.2%) |
| ECOG score at the time of SMM diagnosis, *n* (%) |  |  |  |
| 0 | 44 (27.2%) | 71 (28.3%) | 94 (27.0%) |
| 1 | 15 (9.3%) | 32 (12.7%) | 40 (11.5%) |
| 2 | 3 (1.9%) | 6 (2.4%) | 15 (4.3%) |
| 3 | 0 (0%) | 0 (0%) | 1 (0.3%) |
| Missing | 100 (61.7%) | 142 (56.6%) | 198 (56.9%) |
| Charlson Comorbidity Index, *n* (%) |  |  |  |
| 0 | 113 (69.8%) | 183 (72.9%) | 243 (69.8%) |
| 1 | 8 (4.9%) | 18 (7.2%) | 29 (8.3%) |
| 2 | 34 (21.0%) | 40 (15.9%) | 65 (18.7%) |
| 3–6 | 7 (4.3%) | 10 (4.0%) | 11 (3.2%) |
| Last laboratory value prior to index date, mean (SD) |  |  |  |
| SCr, mg/dL | 1.1 (0.5) | 1.2 (1.0) | 1.2 (0.8) |
| Hemoglobin, g/dL | 12.8 (1.6) | 12.9 (1.9) | 12.7 (1.9) |
| SCa, mg/dL | 9.3 (0.5) | 9.4 (0.6) | 9.4 (0.6) |
| LDH, U/L | 250 (168) | 202 (114) | 205 (113) |

^a^Age at diagnosis.

*ECOG* Eastern Cooperative Oncology Group, *LDH* lactate dehydrogenase, *SCa* serum calcium, *SCr* serum creatinine, *SD* standard deviation, *SMM* smoldering multiple myeloma.
