# Supplementary material for: Real-world characteristics and outcomes of patients with high-risk and non-high-risk smoldering multiple myeloma using the Flatiron Health database
Source: Blood Cancer J. 2024 Dec 5;14(1):215. doi: 10.1038/s41408-024-01170-z (PMC11621302; doi:10.1038/s41408-024-01170-z)
Supplement: Supplementary file 2 — Supplementary Table 2. Baseline characteristics in patients with SMM risk-stratified by IMWG 2020 criteria. [file 41408_2024_1170_MOESM2_ESM.docx]

**Supplementary Table 2.** Baseline characteristics in patients with SMM risk stratified by IMWG 2020 criteria.

| **Characteristic** | **High risk**  ***n* = 103** | **Intermediate risk**  ***n* = 102** | **Low risk**  ***n* = 56** |
| --- | --- | --- | --- |
| Age^a^, years |  |  |  |
| Mean (SD) | 67.3 (9.1) | 68.2 (10.0) | 67.8 (10.8) |
| Median (min, max) | 69.0 (41.0, 82.0) | 70.0 (36.0, 82.0) | 71.5 (36.0, 81.0) |
| Male sex, *n* (%) | 57 (55.3%) | 51 (50.0%) | 23 (41.1%) |
| Race and ethnicity, *n* (%) |  |  |  |
| White | 71 (68.9%) | 63 (61.8%) | 37 (66.1%) |
| Black/African American | 14 (13.6%) | 18 (17.6%) | 9 (16.1%) |
| Hispanic/Latino | 0 (0%) | 0 (0%) | 0 (0%) |
| Asian | 2 (1.9%) | 1 (1.0%) | 2 (3.6%) |
| Other race | 9 (8.7%) | 16 (15.7%) | 7 (12.5%) |
| Missing | 7 (6.8%) | 4 (3.9%) | 1 (1.8%) |
| ECOG score at the time of SMM diagnosis, *n* (%) |  |  |  |
| 0 | 32 (31.1%) | 34 (33.3%) | 24 (42.9%) |
| 1 | 10 (9.7%) | 19 (18.6%) | 5 (8.9%) |
| 2 | 2 (1.9%) | 4 (3.9%) | 2 (3.6%) |
| 3 | 0 (0%) | 0 (0%) | 0 (0%) |
| Missing | 59 (57.3%) | 45 (44.1%) | 25 (44.6%) |
| Charlson Comorbidity Index, *n* (%) |  |  |  |
| 0 | 72 (69.9%) | 75 (73.5%) | 38 (67.9%) |
| 1 | 3 (2.9%) | 13 (12.7%) | 7 (12.5%) |
| 2 | 25 (24.3%) | 12 (11.8%) | 11 (19.6%) |
| 3–6 | 3 (2.9%) | 2 (2.0%) | 0 (0%) |
| Last laboratory value prior to index date, mean (SD) |  |  |  |
| SCr, mg/dL | 1.1 (0.5) | 1.2 (0.9) | 1.3 (1.2) |
| Hemoglobin, g/dL | 12.9 (1.4) | 12.9 (1.9) | 12.8 (1.5) |
| SCa, mg/dL | 9.4 (0.5) | 9.4 (0.6) | 9.4 (0.5) |
| LDH, U/L | 214 (125) | 232 (146) | 192 (91) |

^a^Age at diagnosis.

*ECOG* Eastern Cooperative Oncology Group, *IMWG* International Myeloma Working Group, *LDH* lactate dehydrogenase, *SCa* serum calcium, *SCr* serum creatinine, *SD* standard deviation, *SMM* smoldering multiple myeloma.
